# Connection Between Sleep and Psychological Well-Being in U.S. Army Soldiers

**DOI:** 10.1093/milmed/usad187

**Published:** 2023-06-02

**Authors:** Kristen E Holmes, Nadia Fox, Jemma King, David M Presby, Jeongeun Kim

**Affiliations:** School of Psychology, University of Queensland, St Lucia, QLD 4072, Australia; Performance Science, Whoop Inc., Boston, MA 02215, USA; School of Psychology, University of Queensland, St Lucia, QLD 4072, Australia; School of Psychology, University of Queensland, St Lucia, QLD 4072, Australia; BioPsych Analytics, Tennyson, QLD 4105, Australia; Performance Science, Whoop Inc., Boston, MA 02215, USA; Performance Science, Whoop Inc., Boston, MA 02215, USA

## Abstract

**Introduction:**

The goal of this exploratory study was to examine the relationships between sleep consistency and workplace resilience among soldiers stationed in a challenging Arctic environment.

**Materials and Methods:**

A total of 862 soldiers (67 females) on an Army base in Anchorage, AK, were provided WHOOP 3.0, a validated sleep biometric capture device and were surveyed at onboarding and at the conclusion of the study. Soldiers joined the study from early January to early March 2021 and completed the study in July 2021 (650 soldiers completed the onboarding survey and 210 completed the exit survey, with 151 soldiers completing both). Three comparative analyses were conducted. First, soldiers’ sleep and cardiac metrics were compared against the general WHOOP population and a WHOOP sample living in AK. Second, seasonal trends (summer versus winter) in soldiers’ sleep metrics (time in bed, hours of sleep, wake duration during sleep, time of sleep onset/offset, and disturbances) were analyzed, and these seasonal trends were compared with the general WHOOP population and the WHOOP sample living in AK. Third, soldiers’ exertion, sleep duration, and sleep consistency were correlated with their self-reported psychological functioning. All analyses were conducted with parametric and non-parametric statistics. This study was approved by The University of Queensland Human Research Ethics Committee (Brisbane, Australia) Institutional Review Board.

**Results:**

Because of the exploratory nature of the study, the critical significance value was set at *P *< .001. Results revealed that: (1) Arctic soldiers had poorer sleep consistency and sleep duration than the general WHOOP sample and the Alaskan WHOOP sample, (2) Arctic soldiers showed a decrease in sleep consistency and sleep duration in the summer compared to that in the winter, (3) Arctic soldiers were less able to control their bedroom environment in the summer than in the winter, and (4) sleep consistency but not sleep duration correlated positively with self-report measures of workplace resilience and healthy social networks and negatively with homesickness.

**Conclusions:**

The study highlights the relationship between seasonality, sleep consistency, and psychological well-being. The results indicate the potential importance of sleep consistency in psychological functioning, suggesting that future work should manipulate factors known to increase sleep consistency to assess whether improved sleep consistency can enhance the well-being of soldiers. Such efforts would be of particular value in an Arctic environment, where seasonality effects are large and sleep consistency is difficult to maintain.

## INTRODUCTION

Like most other animals, humans follow a circadian (∼24-hour) cycle in their temperature, hormones, cognitive functioning, and mood. The circadian system coordinates the daily timing of biochemical, physiological, and behavioral processes.^1^ Interference of the circadian system by environmental manipulations has been associated with a host of mental^2–6^ and physical disorders.^7–11^ Most notably for the current research, the stability of bedtime and rise-time is critical for entrainment of the circadian system.^12^

Humans rely on daily patterns in sunlight and temperature to determine sleep-wake cycles through programming of the circadian clocks, creating seasonally shifting but highly consistent sleep schedules.^13^ This form of sleep consistency, whereby people go to bed and wake up at roughly the same time daily, is a determinant of sleep quality and mental health.^14^ Many of the conveniences of modern life, such as constant accessibility of light, food, and the ability to engage in physical activity out of phase with the natural light/dark cycle, can deprive our circadian system of the natural synchronizers on which our ancestors relied.

The average U.S. citizen spends 93% of their life indoors.^15^ Time spent indoors reduces daytime light exposure and increases artificial light exposure at night, resulting in a dampened light/dark signal, the most important synchronizer for the human circadian system.^16,17^ The dampening of this signaling pathway suppresses the natural release of the sleep hormone melatonin, a central part of the body’s sleep-wake cycle.^18^ As a result, people often struggle to fall asleep, wake up during the night, or are unable to sleep as long as they want.^19^ Insufficient sleep, in turn, leads to decreased cognitive performance^20^ and emotional dysregulation.^21^

This disruption of the light/dark cycle is magnified in an Arctic environment, where the increased seasonal variation in sunlight and mental health challenges experienced by soldiers in AK provided an opportunity to interrogate the effects of seasonality on sleep and workplace resilience. According to the U.S. DoD, suicides among active duty service members increased by more than 40% between 2015 and 2020.^22^ At Elmendorf-Richardson Base, Anchorage, AK, USA, where the study was conducted, the suicide rate has doubled.

The goal of the current study was to examine the relationship between sleep-wake consistency, sleep quality, sleep duration, and operationally relevant measures of psychological functioning. The original intent of the study was to enhance sleep consistency via random assignment to a sleep education protocol, but there was no appreciable uptake of the sleep education by those in the experimental condition. Consequently, we leveraged the data collection to conduct an exploratory study of the relationships between natural variation in sleep consistency, workplace resilience, and changing seasons in an Arctic environment.

## MATERIALS AND METHODS

To assess sleep performance, we used a validated sleep biometric capture device (WHOOP 3.0) to measure sleep consistency and overall sleep duration. To assess resilience, we measured heart rate variability (HRV; variability in the beat-to-beat timing when at rest), resting heart rate (RHR), and self-reported measures of workplace resilience. To provide benchmarks for comparison, the biometric data from the soldiers were supplemented with data from an age- and gender-matched cohort of WHOOP users. We also compared the soldiers to a sample of WHOOP users from AK, to control for factors unique to the Arctic environment.

The original experimental design and hypotheses were pre-registered on the Open Science Framework (OSF; https://osf.io/pgy3z/). Because soldiers did not participate in their randomly assigned sleep education protocols, the focus of the “Materials and Methods” and “Results” sections of this article is on measures that allowed us to assess the relationships between natural variation in sleep consistency and workplace resilience. All relevant data and questions are included in the analyses (see Supplemental Online Materials (SOM) for full questionnaire).

Because of the non-normal distribution of some of the variables, all analyses were conducted with both parametric and non-parametric tests, the results of which were functionally identical. Here, we report within-group comparisons conducted with a Wilcoxon signed-rank test and between-group comparisons conducted with a Mann–Whitney U-test. Analyses that include both within-group and between-group comparisons are presented with a two-way mixed-model ANOVA. We also report relationships between variables using Pearson correlations and Ordinary Least Squares (OLS) multiple regression. R packages include “MatchIt”^23^ for matching samples, “stats”^24^ for statistical analyses, and “ggplot2”^25^ and “ggstatsplot”^26^ for visualizations.

### Participants

Approximately one thousand U.S. soldiers based in Anchorage in AK, USA, were invited to take part in the study. A total of 862 soldiers (67 females) chose to participate and were provided WHOOP 3.0, wrist-worn biometric capture devices. When onboarding to the WHOOP application, soldiers were asked if they identify as male, female, non-binary, or chose not to answer. All participants opted to self-identify as male or female. Participants were given links to the survey at the time of onboarding and at the conclusion of the study, with 656 soldiers completing the onboarding survey and 210 soldiers completing the same survey at the study’s conclusion. Soldiers joined the study between January and March 2021 and provided data through July 2021.

### Measures

Responses were provided on 7-point scales with 1 = strongly disagree and 7 = strongly agree, unless otherwise indicated.

### Workplace Resilience

A measure of workplace resilience was created by adapting items from scales assessing job satisfaction, workplace mental health, and additional items based on interviews with soldiers. The resultant six-item questionnaire was as follows: (1) Most days, my work is interesting to me; (2) I know what I want in life and how to achieve it; (3) when I wake up, I am usually excited to get to work; (4) I can usually handle whatever comes my way; (5) our Operating Tempo is hard to sustain (reverse scored); and (6) I believe I can succeed at almost anything I try. The reliability for the scale was adequate at onboarding (alpha = .65) and acceptable at exit (alpha = .76).

### Control Over Sleep Environment/Sleep Habits

The measures of control over the sleeping environment and actual sleep habits were based on research indicating the importance of seven behaviors for sleep quality and duration^27–29^: (1) I can make my room cold if I want to; (2) I can make my room dark if I want to; (3) I can make my room quiet if I want to; (4) I can go to bed and wake up at a similar time each day if I want to; (5) I can control when I eat the last meal of the day if I want to; (6) I can avoid bright lights before bedtime if I want to; and (7) I can put my laptop and phone on night mode before bed if I want to. The reliability for the scale was acceptable at onboarding (alpha = .74) and exit (alpha = .73). These seven items were then rephrased to measure sleep habits (e.g., “I keep my bedroom cold when I’m sleeping”), although the reliability was poor at onboarding (alpha = .43) and exit (alpha = .52), suggesting a disconnect between what soldiers could do and what they actually chose to do during their bedtime routine.

### Work Burnout

The measure of work burnout was adapted from the Maslach burnout inventory. The three-item questionnaire was as follows: (1) Does work energize you? (reversed), (2) does work wear you down?, and (3) do you feel burned out by work? Responses to these items were provided on a 5-point scale with 1 = never and 5 = very often. The reliability of the scale was acceptable at onboarding (alpha = .79) and good at exit (alpha = .83).

### Anxiety

The measure of anxiety was developed through interviews with soldiers. The four-item questionnaire was as follows: (1) Today, I worried a lot; (2) today, I felt jittery; (3) today, I felt judged by my peers; and (4) today, I felt judged by my superior officers. Responses to these items were provided on a 5-point scale with 1 = never and 5 = very often. The reliability of the scale was good at onboarding (alpha = .84) and exit (alpha = .83).

### Homesickness

The two-item measure of homesickness was as follows: (1) How often do you miss your friends/family back home? and (2) how often do you miss being back home? Responses to these items were provided on a 5-point scale with 1 = never and 5 = very often. The reliability of the scale was excellent at onboarding (alpha = .92) and exit (alpha = .92).

### Social Networks

A measure of social networks, developed through interviews with soldiers, was intended to tap the degree to which soldiers socialized with people who had positive traits and habits. The eight-item questionnaire was as follows (the first six items are reverse coded): (1) The people I hang out with do not like it here; (2) the people I hang out with smoke a lot; (3) the people I hang out with drink a lot; (4) the people I hang out with eat a lot of junk food, (5) the people I hang out with play a lot of video games, (6) the people I hang out with stay up late at night, (7) the people I hang out with keep fit, and (8) the people I hang out with are upbeat. The reliability of the scale was acceptable at onboarding (alpha = .77) and good at exit (alpha = .82).

### Social Support

A measure of social support, developed through interviews with soldiers, was intended to tap the degree to which soldiers socialized with people who supported them. The five-item questionnaire was as follows: (1) I can rely on the people I hang out with to help me, (2) the people I hang out with genuinely care about me, (3) the people I hang out with share my values, (4) the people I hang out with push me to improve myself, and (5) I have people in my life who will support me no matter what. The reliability of the scale was good at onboarding (alpha = .82) and exit (alpha = .89).

### Belonging

A measure of belonging was developed to tap the degree to which soldiers felt a bond with their fellow soldiers. The four-item questionnaire was as follows: (1) I feel a bond with my fellow soldiers; (2) I sometimes feel disconnected from the people around me (R); (3) I feel that I belong on this base; and (4) my team feels like family. The reliability of the scale was acceptable at onboarding (alpha = .75) and good at exit (alpha = .81).

### Physiological Measures

Personal wrist-worn biometric capture devices (WHOOP 3.0, Inc., Boston, MA) were worn by participants 24/7 for the duration of the study to provide valid measures of sleep, HRV, RHR, and exertion.^30,31^ Sleep duration is calculated as the sum of light, slow wave sleep (SWS) and rapid eye movement sleep. Sleep Consistency (a proprietary metric of the WHOOP platform adapated from the Sleep Regularity Index),^32^ which calculates the percentage of concordance when individuals are in the same state [asleep vs awake] at different timepoints.Whereas the sleep regularity index compares only two time points 24 hours apart, WHOOP sleep consistency compares sleep onset and offset times over a 4-day interval (e.g., onset today versus onset yesterday and onset today versus the day before), with comparisons of intervals further apart assigned progressively lower weights in calculating sleep consistency scores. Scores are converted and expressed as a percetange on a scale of 0% to 100%, with higher consistency score reflecting lower variability in sleep-wake timing. WHOOP 3.0 also measures calories burned which we call “exertion”. To estimate basal metabolic rate and calories, WHOOP uses a “revised Harris-Benedict Equation”.^33^ Data from the WHOOP 3.0 have been compared to sleep/wake data collected simultaneously using polysomnography, with high levels of agreement between the two methods of assessment.^34^ No reliable effects emerged with HRV or RHR, so they are not discussed further in this article (but see Table I for means).

**TABLE I.** Demographics and Physiological Profile of the U.S. Army Soldiers Stationed in AK, the Age- and Gender-matched WHOOP Users, and WHOOP Users Based in AK

	Army			General WHOOP population			WHOOP Alaskan		
Metric	Male	Female	All	Male	Female	All	Male	Female	All
*N*	795	67	862	795	67	862	544	250	794
Age	27.03 ± 5.94	26.12 ± 5.90	26.96 ± 5.94	25.98 ± 2.88	25.33 ± 3.50	25.93 ± 2.94	34.32 ± 11.13	35.17 ± 10.28	34.59 ± 10.87
Height (m)	1.77 ± 0.07	1.68 ± 0.10	1.76 ± 0.08	1.80 ± 0.07	1.66 ± 0.07	1.79 ± 0.08	1.80 ± 0.09	1.65 ± 0.07	1.75 ± 0.11
Weight (kg)	83.89 ± 11.59	69.80 ± 14.45	82.79 ± 12.42	85.43 ± 13.39	67.65 ± 13.95	84.05 ± 14.24	90.02 ± 17.04	72.45 ± 16.60	84.49 ± 18.76
HRV (rMMSDS)	72.10 ± 30.20	73.66 ± 32.85	72.22 ± 30.40	71.78 ± 28.26	65.45 ± 28.56	71.29 ± 28.32	60.45 ± 29.70	54.67 ± 27.30	58.63 ± 29.07
RHR (BPM)	57.16 ± 6.77	60.47 ± 6.63	57.42 ± 6.82	56.57 ± 7.33	61.91 ± 7.78	56.98 ± 7.50	58.33 ± 7.91	62.06 ± 8.35	59.50 ± 8.23
Exertion (kcal/day)	2325.88 ± 431.81	1743.80 ± 300.87	2280.48 ± 450.84	2465.31 ± 357.55	1816.93 ± 207.91	2414.91 ± 389.08	2502.36 ± 1071.48	1739.11 ±240.63	2262.70 ± 964.83
Sleep duration	6 hours 37 minutes ± 49 minutes	6 hours 52 minutes ± 51 minutes	6 hours 38 minutes ± 49 minutes	6 hours 50 minutes ± 37 minutes	7 hours 12 minutes ± 38 minutes	6 hours 52 minutes ± 37 minutes	6 hours 42 minutes ± 53 minutes	7 hours 8 minutes ± 45 minutes	6 hours 50 minutes ± 52 minutes
Sleep consistency (%)	52.57 ± 12.25	51.64 ± 12.54	52.49 ± 12.27	66.68 ± 8.65	69.10 ± 9.97	66.87 ± 8.78	63.68 ± 11.51	67.47 ± 10.76	64.87± 11.41

All data are shown in mean ± SD. The soldiers showed significantly shorter height, less exertion, sleep duration, and sleep consistency than the matched controls. The soldiers were more likely to be male, younger in age, with higher HRV and lower RHR, and lower sleep duration and consistency than the WHOOP users based in AK. The WHOOP users based in AK were more likely to be male, older, shorter, with lower HRV and exertion, and higher RHR than the general WHOOP population.

Abbreviations: HRV, heart rate variability; RHR, resting heart rate.

### Demographics

Soldiers entered their age, gender, height, and weight into the WHOOP platform when they received the biometric device. Race/ethnicity was not assessed, but soldiers at Elmendorf-Richardson Base are approximately 54% White, 21% Hispanic, 9% African American, 4% Asian, 1% Native American, and 11% others.

## RESULTS

Because of the exploratory nature of this research, we focused on relationships with *P *< .001.

### Comparison 1: U.S. Army Soldiers, the General WHOOP Population, and WHOOP Population in AK

Demographic characteristics and physiological markers are reported in Table I, which provides a comparison between the soldiers, the age- and gender-matched sample, and AK-based WHOOP users. As is evident in Table I, soldiers did not vary substantially from the matched controls in terms of weight, HRV, and RHR, but soldiers showed less exertion (*z *= 6.00, *P < *.001), sleep duration (*z = *7.32, *P < *.001), and sleep consistency (*z = *23.68, *P < *.001) than the matched controls. Compared to the WHOOP users based in AK, soldiers were more likely to be male (*z = *12.25, *P < *.001), younger (*z = *16.54, *P < *.001), with higher HRV (*z = *10.19, *P < *.001), lower RHR (*z = *4.93, *P < *.001), lower sleep duration (*z = *6.32, *P < *.001), and poorer sleep consistency (*z = *19.48, *P < *.001).

### Comparison 2: Seasonal Trends of the Sleep Metrics in Soldiers

Winter data were defined as January 2, 2021, to February 28, 2021, and summer data from May 1, 2021, to July 10, 2021. Preliminary analyses revealed that soldiers with at least 23 days of physiological data during winter and at least 30 days of data during summer provided a good approximation of the full data set (*r’s *≥ .80), providing a sample of 140 soldiers (8 females) who met both criteria. Based on this subset of soldiers with data from both seasons, we extracted a WHOOP-matched cohort and an age-matched WHOOP cohort from AK in a 1:1 ratio.

#### Sleep consistency

**FIGURE 1.**
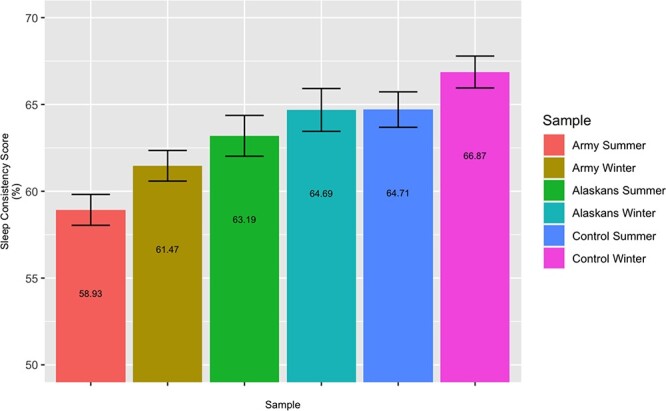
Seasonal differences in sleep consistency. Average sleep consistency of soldiers, the civilians in AK (“Alaskans”), and the age- and gender-matched cohort (“Control”) in the summer and winter. The numbers show the average and the bars represent one SEM.

As is evident in Fig. 1, soldiers showed lower sleep consistency during summer (*z *= 4.61, *P *< .001) and winter (*z *= 4.29, *P *< .001) than the matched cohort. Soldiers also showed lower sleep consistency during summer than winter (*z *= 4.47, *P *< .001; see SOM for a gender breakdown of the analyses). Tentative evidence for the seasonal drop in sleep consistency was also found in the matched cohort (*z *= 2.63, *P* < .01) and in the civilians in AK (*z* = 2.01, *P* = .04), although neither finding met our significance criterion. The magnitude of the seasonal change in sleep consistency did not differ significantly across the three samples (*P*’s > .40).

To investigate if exertion levels throughout the day contributed to the seasonal drop in sleep consistency, we broke the day into three parts and regressed the seasonal change in sleep consistency on the seasonal change in exertion levels in the morning, afternoon, and evening. Only change in evening activity predicted sleep consistency at *P *< .05 and, only among the soldiers, raising the possibility that increased evening activity in the summer may be detrimental to sleep consistency, particularly in AK’s high latitude. Because the finding failed to reach our significance cutoff of *P *< .001, it should be considered tentative and is reported in the SOM.

#### Sleep duration, onset, offset, and disturbances

**FIGURE 2.**
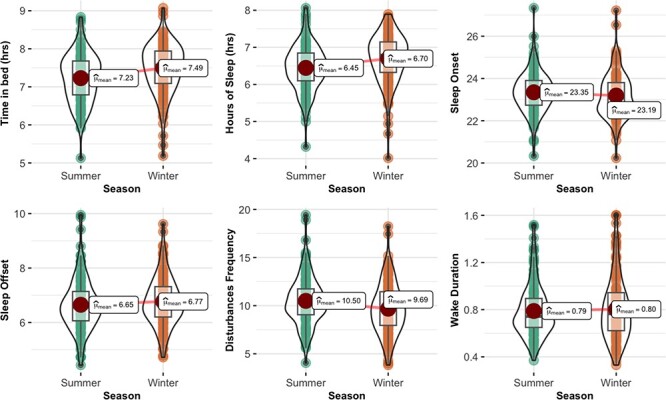
Seasonal differences in time in bed, hours of sleep, sleep onset and offset, disturbances frequency, and wake duration of soldiers seasonal differences in: Top left; time in bed, *P *< .001 (total duration from sleep onset to sleep offset, not including time before falling asleep), top center; hours of sleep, *P *< .001, top right; sleep onset, *P *< .001, bottom left; sleep offset, *P *= .01, bottom center; disturbance frequency, *P *< .001, bottom right; awake duration, *P *= .92 (total duration of sleep disturbances after sleep onset before sleep offset), among U.S. Army soldiers stationed in AK.

As is evident in Fig. 2, the time from when soldiers first fell asleep to when they woke up was approximately 16 minutes less in the summer than in the winter (*z *= 5.95, *P *< .001) resulting in 15 minutes less quality sleep (*z *= 6.49, *P *< .001) and approximately 1 minute less time awake between sleep episodes (*z *= 0.10, *P *= .9). Soldiers woke up about 7 minutes earlier in the summer (*z *= 2.54, *P = *.011) and fell asleep about 10 minutes later (*z *= 3.35, *P *< .001). Soldiers also experienced more disturbances in the summer than that in the winter (*z *= 6.14, *P *< .001). OLS regression revealed that changes in sleep offset (β = .70, *P *< .001) and sleep onset (β = −.75, *P *< .001) predicted the change in sleep duration, whereas the change in disturbance was not a significant predictor (β = .020, *P *= .21). Consistent with these changes, soldiers showed tentative evidence for a decrease in their ability to control their sleep environment in the summer (mean [M] = 2.31, SD = 0.51) compared to winter (M = 2.48, SD = 0.50; *z* = 4.01, *P *< .001). Seasonal distributions of the sleep onset and offset times are presented in the SOM (Fig. S1).

The same trends were observed in age- and-gender-matched civilians in AK with data available in both seasons. They also experienced more disturbances (11.1 versus 10.3; *z *= 4.12, *P *< .001), 23 minutes less sleep (6.64 hours versus 7.02 hours; *z* = 5.50, *P* < .001), and earlier sleep offset (7:20 am versus 7:37 am,*z* = 2.03, *P* = .04) in the summer, but no differences in sleep onset (11:36 pm versus 11:36 pm; *z *= 1.07, *P *= .28; only disturbances and hours of sleep met the significance level of this study). When soldiers were compared to civilians in AK on these metrics, results indicated that soldiers woke up earlier than civilians (*P *< .001), but no other differences emerged.

### Comparison 3: Sleep Consistency, Sleep Duration, Exertion, and Workplace Resilience

**FIGURE 3.**
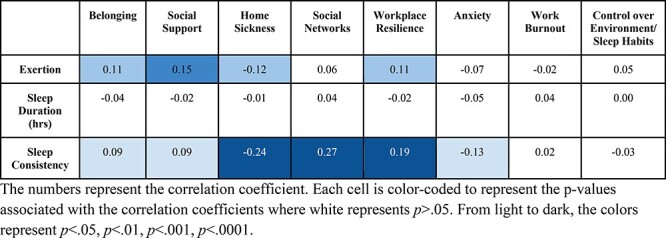
Correlation between exertion, hours of sleep, sleep consistency, and measures of psychological functioning. The numbers represent the correlation coefficient. Each cell is color-coded to represent the *P*-values associated with the correlation coefficients where white represents *P *> .05. From light to dark, the colors represent *P *< .05, *P *< .01, *P *< .001, *P *< .0001.

Pearson correlations were used to examine the relationship between exertion, sleep duration, and sleep consistency during the 14 days before the onboarding survey and the self-report measures of psychological functioning collected at onboarding. As can be seen in Fig. 3, greater sleep consistency was associated with less homesickness, more positive social networks, and greater workplace resilience. Sleep duration, in contrast, did not correlate with any of the self-report measures and exertion only correlated with social support. Nonetheless, tentative support emerged for relationships between sleep consistency and belonging, social support, and anxiety and, to a lesser degree, between exertion and belonging, homesickness, and workplace resilience.

## DISCUSSION

The current findings highlight several key factors that might influence psychological functioning in a particularly challenging Arctic military environment. Sleep consistency was associated with a number of indicators of good psychological functioning (Fig. 3). In particular, greater sleep consistency correlated with self-reports of less homesickness, more positive social networks, and greater workplace resilience. Unfortunately, soldiers’ sleep consistency was substantially lower than that of an age-matched control sample and a sample of Alaskan civilians (Table I and Fig. 1). Although the current findings suggest that seasonality might account for some of the losses in soldiers’ sleep consistency, they do not rule out other potential contributing factors. Given that soldiers in this study engaged in remote in-field training, disrupted sleep schedules from shift work may also play a role in their poor sleep consistency. Although we did not measure or control for shift work directly, Fig. S2 in the SOM suggests that shift work outside normal waking hours was uncommon in this soldier population. Nonetheless, the influence of shift work on Alaskan soldiers’ sleep consistency remains an open question for future research.

Examination of the relationship between sleep consistency and mental and physical health beyond the effects of sleep timing, duration, and quality is rare, but prior evidence raises the possibility of independent effects of sleep consistency. For example, along with self-reported insomnia and nightmares, biometrically assessed sleep consistency emerged as an acute warning sign of suicidal ideation.^35,36^ Along with the current results, such findings highlight the potential utility of sleep consistency as a marker of well-being and suicide risk and thus a potential therapeutic target.

The findings highlight the possibility that AK’s high latitude poses a unique challenge to sleep consistency. The military base where this study was conducted experiences 19.5 hours of sunlight at the height of summer (with twilight for the rest of the night and no actual darkness) and only 5.5 hours of sunlight in the depths of winter. This dramatic change in sunlight and the resultant dampened light/dark signal in summer may impair the establishment of a stable sleep schedule. Consistent with this possibility, soldiers’ sleep consistency was less stable in summer than winter, soldiers got less sleep in summer by virtue of going to bed later and waking up earlier (Fig. 2), and they also reported less control over their bedtime environment. Civilians living in AK also got less sleep in summer than in winter, suggesting that the seasonal challenges of the Alaskan environment are not unique to soldiers.

Although the findings regarding exertion did not pass our stringent significance threshold, there was tentative evidence for the possibility that higher levels of biometrically captured exertion were associated with increased sense of belonging and workplace resilience and less homesickness. These results replicate prior research showing that physical activity is associated with greater overall well-being.^37^

## CONCLUSION

This study highlights the importance of factors at military bases that impact circadian rhythms and the functioning of soldiers. The primary limitation to these findings is that they are cross-sectional and correlational in nature, making it impossible to know whether sleep consistency plays a causal role in creating higher levels of workplace resilience. Feelings of workplace resilience might enhance sleep consistency or a third factor may be responsible for both. Nonetheless, the current findings highlight the potential importance of sleep consistency as a modifiable risk factor in a military setting and provide preliminary evidence that experimental interventions to enhance sleep consistency might improve workplace resilience and other indicators of healthy psychological functioning.

Finally, and importantly, the current study found no effect of sleep duration on any measures of psychological functioning. Extensive research indicates that sleep duration is important, but the current findings highlight the fact that the general recommendation to increase time in bed might be incomplete advice on its own. Rather, the current results suggest that a focus on sleep consistency, in addition to sleep duration, might yield more benefits.

## Supplementary Material

usad187_Supp

## Data Availability

Anonymized data are uploaded to the OSF along with the original survey DOI 10.17605/OSF.IO/PGY3Z (OSF site is currently private but can be made available to the editor and reviewers on request). The WHOOP data are the property of WHOOP (Boston, MA, USA).

## INSTITUTIONAL CLEARANCE

Does not apply.
