# Supplementary material for: Connection Between Sleep and Psychological Well-Being in U.S. Army Soldiers
Source: Mil Med. 2023 Jun 2;189(1-2):e40–8. doi: 10.1093/milmed/usad187 (PMC10824482; doi:10.1093/milmed/usad187)
Supplement: usad187_Supp [file usad187_supp.zip › SOM_Sleep Consistency and Resilience .docx]

**Supplemental material linked to the online version of the paper include:**

**Demographics and Physiological Profile of the age-and-gender-matched WHOOP users and WHOOP users based in Alaska (S1)**

**Sleep Consistency Analysis Breakdown by Gender (S2)**

**Seasonal Trends in Exertion and Sleep Consistency (S3)**

**Seasonal Distribution of Sleep Onset and Offset among Soldiers (Figure S1)**

**Seasonal Trends in Sleep Consistency Breakdown by Gender (Figure S2)**

**Complete Questionnaire (https://osf.io/pgy3z/)**

**Demographics and Physiological Profile of the age-and-gender-matched WHOOP users and WHOOP users based in Alaska (S1)**

WHOOP users based in Alaska were more likely to be male (*z=*12.25, *p<*.001), older (*z=*19.19, *p<*.001), shorter (*z=*7.92, *p<*.001), with lower HRV (*z=*10.12, *p<*.001), lower exertion (*z=*8.20, *p<*.001), and higher RHR (*z=*5.95, *p<*.001) than the age-and-gender-matched Whoop sample.

**Sleep Consistency Analysis Breakdown by Gender (S2)**

Male soldiers showed poorer sleep consistency in summer than winter, *z*=4.30, *p*<.001, but female soldiers did not, *z*=1.30, *p*=.195. Male soldiers also showed poorer sleep consistency than male controls, in both winter, *z*=4.37, *p*<.001, and summer, *z*=4.68, *p*<.001. No differences emerged in sleep consistency between female soldiers and female controls during summer or winter, but the sample size was too small for proper comparison. Due to the small sample size of female soldiers, the seasonal analyses and comparisons with matched controls are underpowered and hence relatively uninformative.

**Seasonal Trends in Exertion and Sleep Consistency (S3)**

Soldiers showed higher exertion levels in the evening during summer (M=501.57, SD=276.43) than winter (M=374.48, SD=281.90), z=4.88, *p*<.001. The matched cohort also showed higher exertion levels in the evening during summer (M=478, SD=223.88) than winter (M=404.14, SD=262.57), z=2.80, p=.005. The seasonal increase in evening activity in soldiers did not differ significantly from the matched control, *z*=1.22, *p*=.22.

To test whether the change in exertion levels in the evening accounted for unique variance in changes in sleep consistency, we conducted an OLS multiple regression with sleep consistency as the criterion and exertion levels in the morning, afternoon, and evening as predictor variables. Among soldiers, change in exertion levels in the evening predicted change in sleep consistency, but not to the level required in our exploratory study (β=-1.55, *p*=.04), whereas changes in exertion levels in the morning (β=-.35, *p*=.64) and in the afternoon (β=.12, *p*=.87) were not significant predictors of sleep consistency. The regression model with the matched controls failed to reveal any significant relationships between exertion levels in the morning, afternoon, or evening and sleep consistency (β=-.02, *p*=.98; β=-0.43, *p*=.74; β=.21, *p*=.86, respectively). These findings raise the possibility that evening activities may be uniquely detrimental to sleep consistency and that summer is a time in which soldiers stationed in Alaska engage in more evening activities.

**FIGURE S1. Seasonal Distribution of Sleep Onset and Offset among Soldiers**

**
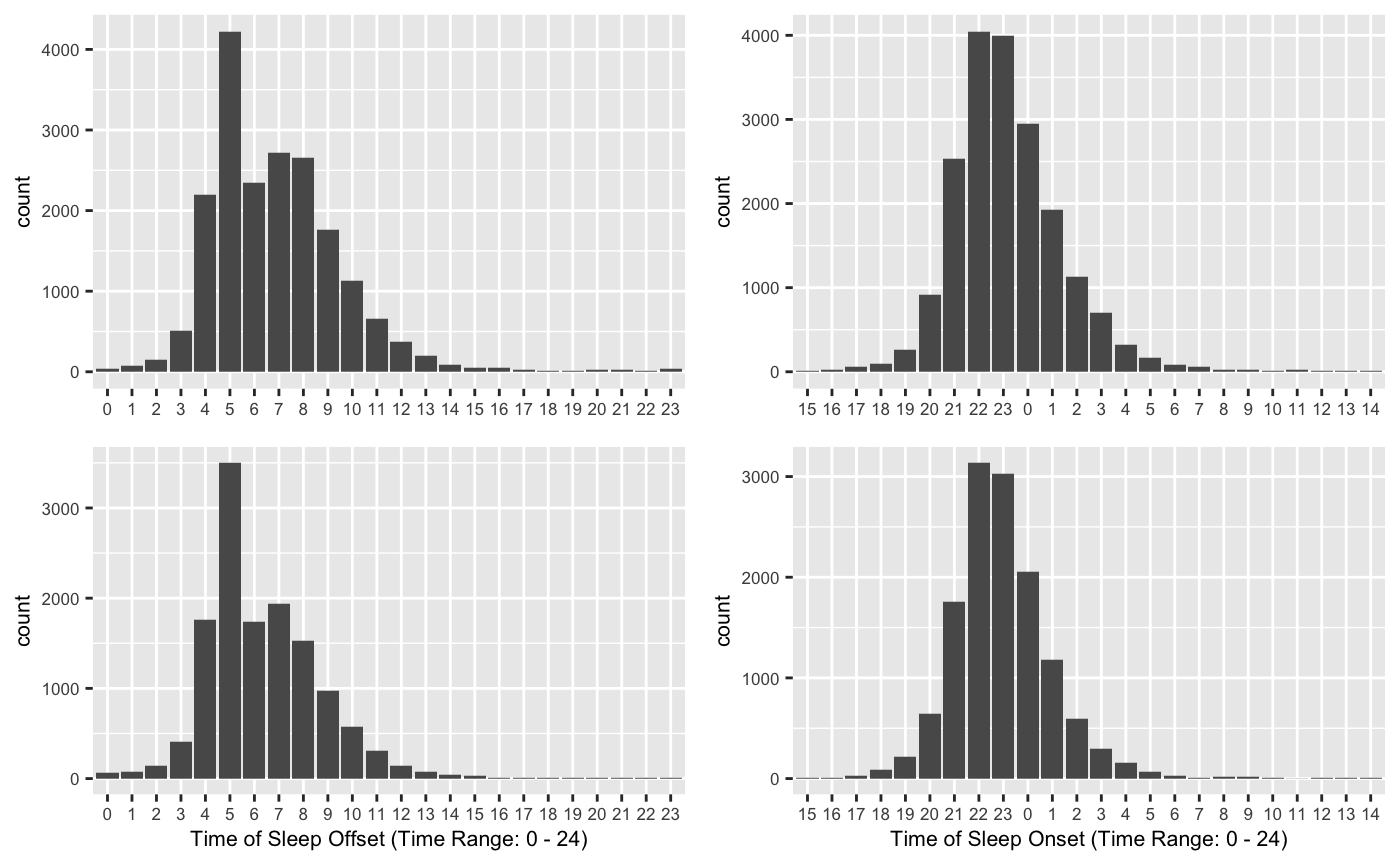
**

Seasonal Distribution of sleep onset and offset times. Time is shown as hours past midnight as the range of 0 - 24. Top Row: Data collected in the winter over 19410 nights across 833 soldiers. Bottom Row: Data collected in the summer over 13378 nights across 442 soldiers. Note that the vast majority of sleep onset and offset times are as expected, suggesting that any shift work in which soldiers were engaged was likely to be primarily during regular waking hours.

**FIGURE S2. Seasonal Trends in Sleep Consistency Breakdown by Gender**

**
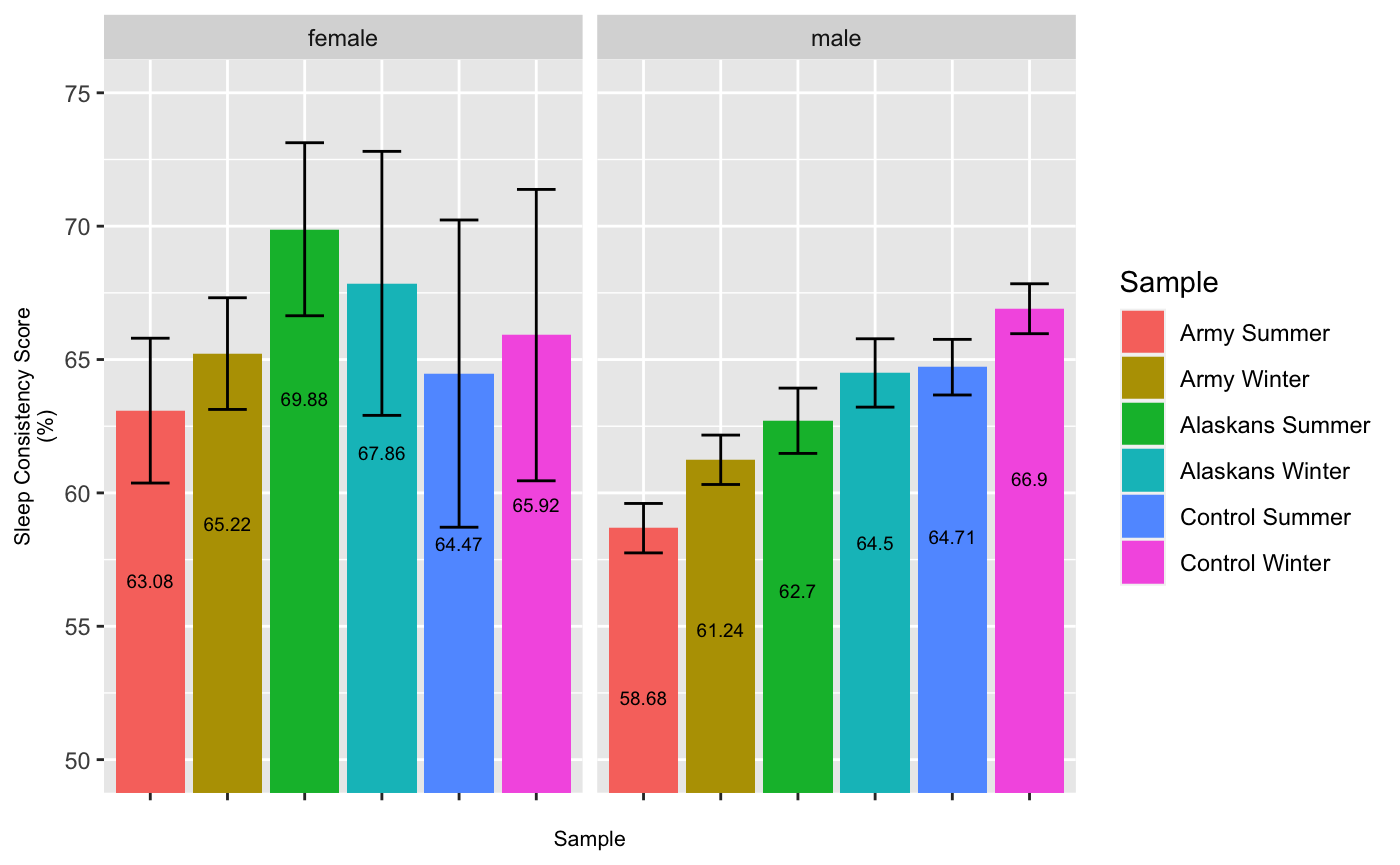
**

Average Sleep Consistency of soldiers, the civilians in Alaska (“Alaskans”), and the age- and gender-matched cohort (“Control”) separated by season and gender. The numbers show the average and the bars represent one standard error of the mean.
